# Adult self-reported and objectively monitored physical activity and sedentary behavior: NHANES 2005–2006

**DOI:** 10.1186/1479-5868-10-126

**Published:** 2013-11-11

**Authors:** John M Schuna, William D Johnson, Catrine Tudor-Locke

**Affiliations:** 1 Pennington Biomedical Research Center, Baton Rouge, LA 70808, USA; 2 Walking Behavior Laboratory, Pennington Biomedical Research Center, Baton Rouge, LA 70808, USA

**Keywords:** Accelerometer, Questionnaire, Sitting, Leisure-time

## Abstract

**Background:**

It remains unclear what people are attempting to communicate, in terms of objectively monitored behavior, when describing their physical activity and sedentary behavior through self-report. The purpose of this study was to examine various objectively monitored accelerometer variables (e.g., moderate-to-vigorous physical activity [MVPA], steps/day, sedentary time, etc.) across categories of self-reported MVPA (< 150 vs. ≥ 150 minutes/week), usual occupational/domestic activity (UODA; “mostly sitting” vs. “stand, walk, lift, or carry”), and leisure-time sedentary behavior (LTSB; ≥ 3 vs. < 3 hours/day) in a nationally representative sample of U.S. adults (≥ 20 years).

**Methods:**

This is a secondary analysis of 3,725 participants from the 2005–2006 National Health and Nutrition Examination Survey (NHANES) who provided relevant questionnaire responses and ≥ 1 day of valid accelerometer data. Descriptive statistics were computed for various objectively monitored accelerometer variables across categories of self-reported MVPA, UODA, and LTSB. Pairwise comparisons were conducted to examine differences in objectively monitored behavior between categories of self-reported MVPA, UODA, and LTSB.

**Results:**

On average, adults reporting compliance with physical activity guidelines (≥ 150 minutes/week of MVPA) accumulated more objectively measured physical activity and similar amounts of sedentary time relative to those reporting not achieving guidelines. Adults reporting their daily UODA as “mostly sitting” or accruing ≥ 3 hours/day of LTSB accumulated less objectively monitored physical activity and more sedentary time than those who described their UODA as “stand, walk, lift, or carry” or accrued < 3 hours/day of LTSB. The most active cross-classified category (7,935 steps/day; ≥ 150 minutes/week of self-reported MVPA, “stand, walk, lift, or carry” UODA, and < 3 hours/day of LTSB) accumulated more than twice as many daily steps as the least active cross-classified category (3,532 steps/day; < 150 minutes/week of self-reported MVPA, “mostly sitting” UODA, and ≥ 3 hours/day of LTSB).

**Conclusions:**

A number of objectively monitored physical activity indicators varied significantly between self-reported MVPA, UODA, and LTSB categories, while objectively monitored sedentary time only varied between UODA and LTSB categories. Cross-classifications of self-reported MVPA, UODA, and LTSB responses depict a greater range of physical activity than viewing dichotomous responses for these variables one-at-a-time.

## Background

Self-reported physical activity and sedentary behavior are independently associated with a number of adverse health outcomes, including mortality [1,2]. Self-reported physical activity is typically captured by a series of questions that identify frequency and duration of engagement in activities that are of at least moderate intensity. In a review of the extant literature, Reimers, Knapp, and Reimers [2] conservatively estimated that the net increase in life expectancy with regular physical activity is approximately 2–4 years, but is plausibly even greater than this because of the known multiple positive influences of physical activity on most major risk factors for mortality. Indeed, the impact of physical inactivity on life expectancy may be on par with that expected of smoking or obesity [3].

Self-reported sedentary behavior is typically captured by general responses to global questions about total daily sitting behaviors and/or time spent in specific sedentary behaviors like leisure-time television watching or computer use [4]. Katzmarzyk and Lee [1] demonstrated that U.S. population life expectancy would be 2.00 years higher if adults reduced their usual sitting to < 3 hours/day and 1.38 years higher if they reduced their television watching behaviors to < 2 hours/day. Although the limitations of self-reported behaviors are well known [5], it must be acknowledged that some representations of self-perceived behaviors (both physical activity and sedentary behaviors) are indeed predictive of unfavorable and undesirable life consequences [1,2].

It is not clear, however, what people are actually attempting to communicate (in terms of objectively monitored behavior) when they describe themselves as achieving public health guidelines for time in moderate-to-vigorous physical activity (MVPA), usually sitting throughout the day (usual occupational/domestic activity [UODA]), or engaging in large amounts of leisure-time sedentary behaviors (LTSB; television watching, leisure-time computer use). As a single illustrative research question, does self-reported usual sitting translate to objectively monitored sedentary time, reduced time in MVPA, fewer steps/day, a lower peak cadence, etc.? Therefore, the purpose of this analysis of the 2005–2006 National Health and Nutrition Examination Survey (NHANES) physical activity monitor (PAM) component is to examine a number of objectively monitored accelerometer variables (e.g., time spent in MVPA, steps/day, sedentary behavior, etc.) across individual and cross-classified categories of self-reported MVPA, UODA, and LTSB.

## Methods

### NHANES 2005–2006

NHANES is the nation’s primary source of objective health data and has been implemented continuously since 1999, enrolling approximately 10,000 people in each two-year assessment cycle. The PAM sub-component introduced accelerometry to NHANES in 2003 to objectively assess each participant’s physical activity patterns. The data are publically available; however, the 2005–2006 NHANES uniquely released accelerometer-determined step data in addition to activity counts/minute data, making it a more comprehensive data set of objective physical activity and sedentary behaviors than the original 2003–2004 NHANES PAM dataset.

The National Center for Health Statistics (NCHS) Ethics Review Board approved all survey protocols and participants provided informed consent before partaking in the study.

### Participants

NHANES participants are routinely selected using a complex, multistage probability design which results in a nationally representative sample of the U.S. civilian, non-institutionalized population. Following household interviews, participants are invited to undergo a 3–4 hour health examination in an NHANES mobile examination center. In 2005–2006, participants ≥ 6 years of age, who reported no walking impairments during the health examination, were invited to wear an accelerometer for 7 days. Participants were compensated $40 for their commitment and for returning accelerometers via prepaid mail. Only data from participants ≥ 20 years of age were considered in the current analysis.

### Measures

#### ***Self-reported physical activity and sedentary behavior***

A physical activity questionnaire was administered by a trained technician during the NHANES household interview component. Participants were asked a series of questions regarding their transportation, household/domestic, and leisure-time physical activity and sedentary behavior.

Several questions were used to assess participants’ engagement in MVPA during the past 30 days related to transportation, household/domestic tasks, and leisure-time activities. All physical activity during transportation and household/domestic tasks was assumed to be of at least moderate-intensity [6,7]. Transportation and household/domestic tasks were evaluated with two questions: 1) “Over the past 30 days, have you walked or bicycled as part of getting to and from work, or school, or to do errands?” and, 2) “Over the past 30 days, did you do any tasks in or around your home or yard for at least 10 minutes that required moderate or greater physical effort?” Participants who answered “Yes” to either question were asked to report the frequency and duration of these activities. Moderate and vigorous leisure-time physical activity were evaluated with two additional questions: 1) “Over the past 30 days, did you do moderate activities for at least 10 minutes that cause only light sweating or a slight to moderate increase in breathing or heart rate?” and 2) “Over the past 30 days, did you do any vigorous activities for at least 10 minutes that caused heavy sweating, or large increases in breathing or heart rate?” Participants who answered “Yes” to either question were asked to specify the activities they engaged in and the usual duration and frequency of these activities.

A single question was used to evaluate participants’ average UODA each day. Participants were asked “Please tell me which of these four sentences best describes your usual daily activities?” For additional clarification, participants were instructed that “Daily activities may include your work, housework if you are a homemaker, going to and attending classes if you are a student, and what you normally do throughout a typical day if you are a retiree or unemployed.” Responses included: 1) “sit during the day and do not walk about very much,” 2) “stand or walk about quite a lot during the day, but do not have to carry or lift things very often,” 3) “lift or carry light loads, or have to climb stairs or hills often,” and 4) “do heavy work or carry heavy loads.”

Two questions were used to assess participants’ daily screen-based LTSB. Specifically, participants were asked: 1) “Over the past 30 days, on average how many hours per day did you sit and watch TV or videos outside of work?” and, 2) “Over the past 30 days, on average about how many hours per day did you use a computer or play computer games outside of work?” Response options for both questions included: 1) “none,” 2) “less than 1 hour,” 3) “1 hour,” 4) “2 hours,” 5) “3 hours,” 6) “4 hours,” and 7) “5 hours or more.”

#### ***Objectively measured physical activity and sedentary behavior***

Physical activity and sedentary behavior were objectively assessed using the ActiGraph accelerometer (model 7164; ActiGraph LLC, Pensacola, FL). Participants were asked to wear the device on their right hip during all waking hours for 7 days and instructed to remove it only for bathing or swimming. The accelerometer was secured to the body by means of an elastic belt. Accelerometers were pre-programmed to record data in 1 minute intervals.

### Data treatment

#### ***Self-reported physical activity and sedentary behavior***

Participant responses to the transportation, household/domestic tasks, and leisure-time physical activity queries were translated to minutes/week of self-reported MVPA [7]. Each participant’s combined weekly duration of self-reported MVPA was grouped into one of two categories (< 150 or ≥ 150 minutes/week) based upon their achievement of current physical activity guidelines [8].

As previously described [9], an overall UODA variable was created by grouping participant responses to the single question evaluating daily average occupational physical activity into two distinct categories: “mostly sitting” or “stand, walk, lift, or carry.”

A composite LTSB variable was created by combining responses from the two screen-based sedentary behavior questions similar to a previous investigation by Sisson and colleagues [9]. Reported values were collapsed into two categories (≥ 3 or < 3 hours/day) to facilitate comparisons across varying levels of LTSB and to maintain sufficient sample sizes for all analyses.

Participants’ data were further grouped into one of eight possible cross-classifications of self-reported MVPA, UODA, and LTSB. This was done to provide finer resolution in describing the objectively monitored activity of participants categorized by different combinations of the abovementioned self-reported variables.

#### ***Objectively measured physical activity and sedentary behavior***

A single data file containing consecutively ordered minute-by-minute accelerometer data (activity counts and steps) from all PAM participants was publicly released in June 2008 [10]. Indicator variables were added to the data file by NHANES staff identifying each accelerometer’s calibration status upon return (calibrated vs. not calibrated) and the reliability of each participant’s accelerometer data (reliable vs. not reliable). Examples of data flagged as not reliable were records containing > 10 minutes with 1) 32,767 activity counts/minute (maximum saturation value for the ActiGraph 7164), 2) 0 steps and > 250 activity counts/minute, and 3) > 200 steps/minute (Captain Richard P. Troiano, personal communication).

Accelerometer data were further screened to identify periods of wear and non-wear time using a publicly available SAS macro developed by the National Cancer Institute (NCI) [11]. Non-wear time was identified as any interval of at least 60 consecutive minutes of 0 activity counts, with allowance for up to 2 consecutive minutes of activity counts between 0 and 100 [12]. Daily wear time was derived by subtracting non-wear time from 1,440 minutes. Only those days with 10 or more hours of wear time were retained for analyses [12].

Accelerometer-determined activity counts and step data were used to derive a series of physical activity and sedentary behavior indicator variables [12-19]. All calculated variables and their operationalized definitions are presented in Table 1. A similar table has been previously published [16]; however, Table 1 displays a broader array of derived variables. We modified another NCI provided SAS macro to compute all the accelerometer-determined variables listed in Table 1[11].

**Table 1:** Accelerometer-determined physical activity and sedentary behavior variable definitions

**Variable**	**Definitions**
	Physical activity monitor (PAM) compliance indicator*
Accelerometer wear time	1,440 minutes minus non-wear time [12].
	Volume indicators*
	***Steps***
Uncensored steps/day	Total raw steps accumulated over 1,440 minutes [19].
Censored steps/day	Total steps accumulated over 1,440 minutes after censoring steps accumulated at intensities < 500 activity counts/minute [19].
	***Activity counts***
Activity counts/day	Total activity counts accumulated over 1,440 minutes [16].
	Rate indicators*
	***Steps***
Uncensored steps/minute	Total raw steps accumulated over 1,440 minutes, divided by accelerometer wear time [16].
Censored steps/minute	Total steps accumulated over 1,440 minutes after censoring steps taken at intensities < 500 activity counts/minute, divided by accelerometer wear time [16].
Peak 1-minute cadence	Steps/minute recorded for the highest single minute in a day [17].
Peak 30-minute cadence	Average steps/minute recorded for the 30 highest, but not necessarily consecutive, minutes in a day [17].
	***Activity counts***
Activity counts/minute	Total activity counts accumulated over 1,440 minutes, divided by accelerometer wear time [12].
	Time indicators*
	***Cadence***
Non-movement	Total minutes at 0 steps/minute during valid wear time [18].
Incidental movement	Total minutes at 1–19 steps/minute [18].
Sporadic movement	Total minutes at 20–39 steps/minute [18].
Purposeful steps	Total minutes at 40–59 steps/minute [18].
Slow walking	Total minutes at 60–79 steps/minute [18].
Medium walking	Total minutes at 80–99 steps/minute [18].
Brisk walking	Total minutes at 100–119 steps/minute [18].
Faster locomotion	Total minutes ≥ 120 steps/minute [18].
Any movement	Total minutes > 0 steps/minute [18].**
Non-incidental movement	Total minutes > 19 steps/minute [18].**
	***Activity intensity***
Sedentary time	Total minutes < 100 activity counts/minute [15].
Low intensity	Total minutes at 100–499 activity counts/minute [16].
Light intensity	Total minutes at 500–2,019 activity counts/minute [19].
Lifestyle intensity	Total minutes at 760–2,019 activity counts/minute [13].
Moderate intensity	Total minutes at 2,020-5,998 activity counts/minute [12].
Vigorous intensity	Total minutes ≥ 5,999 activity counts/minute [12].
Moderate-to-vigorous intensity	Total minutes ≥ 2,020 activity counts/minute [12].
Moderate-to-vigorous intensity in 10 minute bouts	Total minutes ≥ 2,020 activity counts/minute accumulated in modified 10 minute bouts [12].†
	Breaks in sedentary time*
Transitions/day	Number of occurrences where activity counts rose from < 100 activity counts in one minute to ≥ 100 activity counts in the subsequent minute [14].

*Values averaged over valid days (≥ 10 hours/day of wear time).

**Variables not previously defined but derived from previously published variable definitions [18].

†10 or more consecutive minutes ≥ 2,020 activity counts/minute, with allowance for 1–2 minutes < 2,020 activity counts/minute.

#### ***Analytic sample***

NHANES self-reported MVPA, UODA, or LTSB data were available for 4,979 men and women ≥ 20 years of age. For each analysis conducted herein, we excluded participants who refused or did not know how to answer questions relevant to self-reported MVPA (n = 2), UODA (n = 4), or LTSB (n = 5), or with missing frequency or duration values needed to calculate self-reported MVPA (n = 18). We further excluded participants with missing accelerometer data (n = 601) and those with accelerometer data that NHANES-designated as unreliable (n =170) or with accelerometers found to be out of calibration upon return (n = 183). Participants who did not accumulate at least one valid day of accelerometer wear time were also excluded (n = 271). The use of a single-day as the minimum criteria for valid wear has been previously justified [19]. In total, the analytic sample consisted of 3,725 adults. Sample frequencies for all gender, age, and ethnicity categories are presented in Table 2.

**Table 2:** Sample frequencies for 2005–2006 NHANES adults with valid accelerometer and physical activity questionnaire data*

**Gender and age group (years)**	**Total**	**Non-Hispanic Whites**	**Non-Hispanic Blacks**	**Mexican American**	**Other Hispanic**	**Other race – including multiracial**
Men						
All	1,770	898	401	360	51	60
20-29	295	110	73	93	13	6
30-39	295	127	69	66	15	18
40-49	322	144	76	78	9	15
50-59	252	155	56	31	3	7
60-69	273	120	78	60	5	10
70-79	203	131	42	24	4	2
80+	130	111	7	8	2	2
Women						
All	1,955	955	446	402	60	92
20-29	432	192	79	126	17	18
30-39	340	130	84	82	15	29
40-49	336	146	95	64	11	20
50-59	270	145	70	37	10	8
60-69	284	132	74	62	5	11
70-79	167	110	32	22	1	2
80+	126	100	12	9	1	4

*Restricted to participants with ≥ 10 hours of accelerometer wear time on at least a single day and complete physical activity questionnaire data (N = 3,725).

### Statistical analysis

All statistical analyses were conducted using procedures for sample survey data in SAS (version 9.3; SAS Institute, Cary, NC) to account for the complex, multistage probability design used in NHANES. All analyses included sample weights to account for oversampling and nonresponse to provide nationally representative results. Descriptive statistics were calculated for each accelerometer-determined variable among the entire sample and across grouping factors of self-reported MVPA, UODA, and LTSB. Standard errors were derived using Taylor series linearization. Accelerometer-determined variables were compared between categories of self-reported MVPA (< 150 vs. ≥ 150 minutes/week), UODA (“mostly sitting” vs. “stand, walk, lift, or carry”), and LTSB (≥ 3 vs. < 3 hours/day) using two-sided *t*-tests with 15 *df*. Because of the large number of *t-*tests conducted, a Bonferroni-correction was applied to control the global type I error rate at α = 0.05 for the 28 comparisons within the 3 separate analyses for self-reported MVPA, UODA, and LTSB. Thus, statistical significance was defined as p ≤ 0.05/28 = 0.0018 for each set of 28 comparisons.

## Results

Overall sample means for all accelerometer-determined variables are presented in Table 3. On average, participants wore the accelerometer 14.0 ± 0.1 hours/day during the PAM protocol. Table 4 presents means of all accelerometer-determined variables by categories of self-reported MVPA (< 150 vs. ≥ 150 minutes/week), UODA (“mostly sitting” vs. “stand, walk, lift, or carry”), and LTSB (≥ 3 vs. < 3 hours/day).

**Table 3:** Mean values for accelerometer-determined variables in 2005–2006 NHANES adults (N = 3,725)

**Variable**	**Mean (SE)**	**95% CI**
Accelerometer wear time (hours/day)	14.0 (0.1)	13.9-14.2
**Volume indicators**		
***Steps (steps/day)***		
Uncensored steps/day	9,685 (107)	9,457-9,912
Censored steps/day	6,549 (106)	6,324-6,774
***Activity counts (counts/day)***		
Activity counts/day	269,107 (3,236)	262,209-276,006
**Rate indicators**		
***Steps (steps/minute)***		
Uncensored steps/minute	11.5 (0.1)	11.3-11.7
Censored steps/minute	7.7 (0.1)	7.5-8.0
Peak 1-minute cadence	100.8 (0.6)	99.6-102.0
Peak 30-minute cadence	71.6 (0.7)	70.0-73.2
***Activity counts (counts/minute)***		
Activity counts/minute	318.1 (3.0)	311.8-324.5
**Time indicators**		
***Cadence (minutes/day)***		
Non-movement	288.9 (2.2)	284.2-293.7
Incidental movement	383.4 (2.1)	378.9-387.8
Sporadic movement	102.1 (1.4)	99.0-105.2
Purposeful steps	38.9 (0.7)	37.4-40.4
Slow walking	15.5 (0.3)	14.8-16.3
Medium walking	7.5 (0.3)	7.0-8.1
Brisk walking	5.0 (0.3)	4.3-5.7
Faster locomotion	1.5 (0.1)	1.3-1.8
Any movement	554.0 (3.0)	547.7-560.3
Non-incidental movement	170.6 (2.2)	165.8-175.4
***Activity intensity (minutes/day)***		
Sedentary time	478.9 (2.6)	473.4-484.4
Low intensity	200.0 (1.5)	196.8-203.1
Light intensity	141.3 (1.8)	137.4-145.1
Lifestyle intensity	87.8 (1.2)	85.1-90.4
Moderate intensity	21.9 (0.6)	20.6-23.2
Vigorous intensity	0.9 (0.1)	0.7-1.0
Moderate-to-vigorous intensity	22.8 (0.7)	21.3-24.2
Moderate-to-vigorous intensity in 10 minute bouts	6.1 (0.4)	5.3-6.9
**Breaks in sedentary time**		
Transitions/day	91.5 (0.4)	90.6-92.5

Values weighted to provide nationally representative estimates.

**Table 4:** Self-reported MVPA, UODA, LTSB, and accelerometer-determined variables in 2005–2006 NHANES adults

	**MVPA**		**UODA**		**LTSB**	
**Variable**	**< 150 minutes/week**	**≥ 150 minutes/week**	**Mostly sitting**	**Stand, walk, lift, or carry**	**≥ 3 hours/day**	**< 3 hours/day**
N	1,736	1,989	849	2,876	1,944	1,781
Accelerometer wear time (hours/day)	14.0 (0.1)	14.1 (0.1)	13.9 (0.1)	14.1 (0.1)	13.9 (0.1)	14.2 (0.1)
**Volume indicators**						
***Steps (steps/day)***						
Uncensored steps/day	8,773 (120)	10,319 (149)^a^	7,657 (156)	10,287 (120)^b^	8,970 (134)	10,437 (117)^c^
Censored steps/day	5,643 (109)	7,178 (139)^a^	4,909 (144)	7,035 (117)^b^	5,948 (131)	7,181 (101)^c^
***Activity counts (counts/day)***						
Activity counts/day	238,328 (3,819)	290,513 (4,144)^a^	212,469 (5,535)	285,915 (3,431)^b^	247,136 (4,580)	292,251 (2,956)^c^
**Rate indicators**						
***Steps (steps/minute)***						
Uncensored steps/minute	10.4 (0.1)	12.2 (0.1)^a^	9.1 (0.2)	12.2 (0.1)^b^	10.7 (0.1)	12.2 (0.1)^c^
Censored steps/minute	6.7 (0.1)	8.5 (0.1)^a^	5.8 (0.2)	8.3 (0.1)^b^	7.1 (0.1)	8.4 (0.1)^c^
Peak 1-minute cadence	94.3 (0.7)	105.3 (0.7)^a^	96.1 (1.0)	102.2 (0.6)^b^	97.4 (0.9)	104.4 (0.7)^c^
Peak 30-minute cadence	64.0 (0.6)	76.8 (1.0)^a^	65.9 (1.2)	73.3 (0.7)^b^	68.2 (1.0)	75.2 (0.8)^c^
***Activity counts (counts/minute)***						
Activity counts/minute	282.7 (4.8)	342.8 (3.5)^a^	252.6 (6.0)	337.6 (3.0)^b^	294.8 (4.9)	342.7 (3.6)^c^
**Time indicators**						
***Cadence (minutes/day)***						
Non-movement	297.7 (3.2)	282.8 (3.1)	328.9 (5.2)	277.0 (1.9)^b^	302.1 (4.0)	275.0 (2.5)^c^
Incidental movement	385.0 (2.4)	382.3 (2.1)	376.7 (4.5)	385.4 (2.3)	375.0 (2.8)	392.2 (2.1)^c^
Sporadic movement	98.3 (1.7)	104.7 (1.7)	78.1 (1.8)	109.2 (1.4)^b^	96.0 (1.5)	108.5 (1.6)^c^
Purposeful steps	35.1 (0.8)	41.5 (0.9)^a^	27.8 (1.0)	42.2 (0.7)^b^	35.4 (0.7)	42.6 (0.9)^c^
Slow walking	13.4 (0.3)	17.0 (0.5)^a^	10.6 (0.5)	17.0 (0.4)^b^	13.9 (0.4)	17.3 (0.4)^c^
Medium walking	6.1 (0.2)	8.5 (0.4)^a^	5.6 (0.3)	8.1 (0.3)^b^	6.8 (0.3)	8.3 (0.3)^c^
Brisk walking	3.2 (0.2)	6.3 (0.5)^a^	4.3 (0.4)	5.2 (0.3)	4.6 (0.4)	5.5 (0.4)
Faster locomotion	0.5 (0.1)	2.2 (0.2)^a^	1.7 (0.3)	1.5 (0.1)	1.1 (0.1)	2.0 (0.2)
Any movement	541.7 (3.9)	562.5 (3.4)^a^	504.7 (6.2)	568.6 (3.1)^b^	532.8 (4.0)	576.3 (3.2)^c^
Non-incidental movement	156.7 (2.7)	180.3 (2.8)^a^	128.0 (3.0)	183.2 (2.4)^b^	157.8 (2.4)	184.1 (2.6)^c^
***Activity intensity (minutes/day)***						
Sedentary time	488.9 (3.6)	471.9 (3.6)	527.7 (4.3)	464.4 (2.7)^b^	489.3 (4.0)	467.9 (3.1)^c^
Low intensity	200.9 (2.1)	199.3 (1.6)	182.3 (2.7)	205.2 (1.7)^b^	194.3 (2.0)	205.9 (1.8)^c^
Light intensity	132.2 (2.2)	147.6 (2.2)^a^	106.5 (2.5)	151.6 (1.7)^b^	131.3 (1.9)	151.8 (2.0)^c^
Lifestyle intensity	80.3 (1.6)	92.9 (1.6)^a^	64.3 (1.8)	94.7 (1.2)^b^	80.2 (1.4)	95.7 (1.3)^c^
Moderate intensity	17.1 (0.5)	25.2 (0.9)^a^	16.2 (0.8)	23.6 (0.7)^b^	19.3 (0.8)	24.6 (0.7)^c^
Vigorous intensity	0.3 (0.1)	1.3 (0.1)^a^	1.0 (0.2)	0.9 (0.1)	0.7 (0.1)	1.1 (0.1)
Moderate-to-vigorous intensity	17.4 (0.6)	26.5 (1.0)^a^	17.2 (0.9)	24.5 (0.7)^b^	20.0 (0.9)	25.7 (0.8)^c^
Moderate-to-vigorous intensity in 10 minute bouts	3.2 (0.2)	8.2 (0.6)^a^	5.0 (0.6)	6.4 (0.4)	5.1 (0.5)	7.2 (0.5)
**Breaks in sedentary time**						
Transitions/day	91.3 (0.6)	91.7 (0.5)	89.0 (0.9)	92.3 (0.4)^b^	89.3 (0.6)	93.9 (0.5)^c^

Values weighted to provide nationally representative estimates and presented as mean (SE). MVPA = moderate-to-vigorous physical activity; UODA = usual occupational/domestic activity; LTSB = leisure-time sedentary behavior.

^a^Significantly different from < 150 minutes/week of MVPA at p < 0.0018.

^b^Significantly different from “mostly sitting” UODA at p < 0.0018.

^c^Significantly different from ≥ 3 hours/day of LTSB at p < 0.0018.

In comparison to participants who reported engaging in < 150 minutes/week of MVPA, all objectively monitored volume and rate indicators of activity were significantly higher among participants reporting ≥ 150 minutes/week of MVPA. No significant differences in accelerometer wear time, non-movement, incidental movement, sporadic movement, sedentary time, or time in low intensity activity were found between categories of self-reported MVPA. However, participants reporting ≥ 150 minutes/week of MVPA had significantly higher means for all other objectively monitored time indicators of physical activity in comparison to those reporting < 150 minutes/week. No significant differences were observed in breaks in sedentary time between participants reporting < 150 minutes/week of MVPA and those reporting ≥ 150 minutes/week.

All volume and rate indicators of activity were significantly lower among participants reporting their UODA as “mostly sitting” than in those who reported “stand, walk, lift, or carry.” No significant differences in accelerometer wear time, incidental movement, brisk walking, faster locomotion, time in vigorous intensity activity, or time in moderate-to-vigorous intensity activity in modified 10 minute bouts were observed between categories of UODA. Non-movement and sedentary time were significantly higher among participants reporting their UODA as “mostly sitting” than in those reporting “stand, walk, lift, or carry.” However, all other time indicators of activity which were significantly different between UODA categories were higher among participants in the “stand, walk, lift, or carry” group. Additionally, participants reporting their UODA as “mostly sitting” had fewer breaks in sedentary time than those who reported “stand, walk, lift, or carry.”

Similar to the MVPA and UODA analyses, all volume and rate indicators of activity were significantly different between LTSB categories, with the highest values observed among participants accumulating < 3 hours/day of LTSB. No significant differences in wear time, brisk walking, faster locomotion, time in vigorous intensity activity, or time in moderate-to-vigorous intensity activity in modified 10 minute bouts were observed between LTSB categories. Non-movement and sedentary time were significantly higher among participants accumulating ≥ 3 hours/day of LTSB compared to those accumulating < 3 hours/day. All other time indicators with significant differences between LTSB categories were higher in the < 3 hours/day group. Breaks in sedentary time were also significantly lower among participants accumulating ≥ 3 hours/day of LTSB than those accumulating < 3 hours/day.

**Figure 1:**
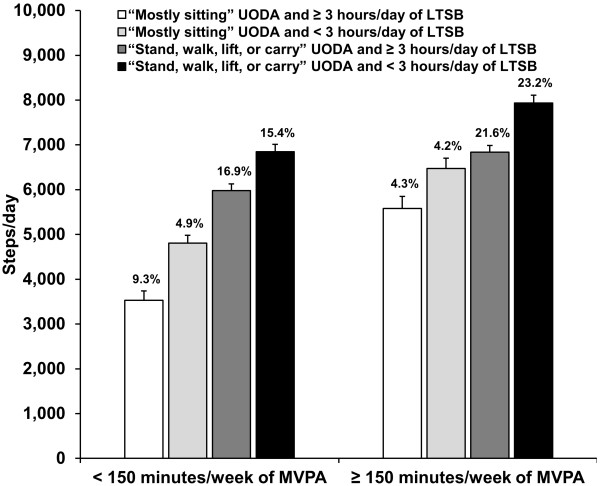
**Means for accelerometer-determined censored steps/day among cross-classifications of self-reported MVPA, UODA, and LTSB.** Values above each bar represent the sample percentage within each cross-classification (N = 3,725). Error bars represent standard error. MVPA = moderate-to-vigorous physical activity; UODA = usual occupational/domestic activity; LTSB = leisure-time sedentary behavior.

Means for censored steps/day within cross-classifications of self-reported MVPA, UODA, and LTSB are displayed in Figure 1 (means for all other accelerometer-determined variables are presented in the Additional file 1: Table S1). Among the eight cross-classifications, participants reporting < 150 minutes/week of MVPA, “mostly sitting” for their UODA, and ≥ 3 hours/day of LTSB were the least active (3,532 ± 206 steps/day). Conversely, participants reporting ≥ 150 minutes/week of MVPA, “stand, walk, lift, or carry” for their UODA, and < 3 hours/day of LTSB were the most active grouping (7,935 ± 173 steps/day).

## Discussion

A number of objectively monitored physical activity indicators varied significantly between categories of self-reported MVPA, UODA, and LTSB, while objectively monitored sedentary time only varied significantly between categories of UODA and LTSB. Specifically, adults reporting compliance with public health guidelines for time spent in self-reported MVPA (≥ 150 minutes/week) were more physically active (objectively determined, multiple volume and rate indicators) but did not engage in less sedentary behavior (also objectively determined) than those not achieving the guidelines (< 150 minutes/week). Adults describing their UODA as “stand, walk, lift, or carry” demonstrated higher objectively monitored values for a number of physical activity indicators *and* less sedentary time than those reporting their UODA as “mostly sitting.” Similarly, adults reporting LTSB accumulations of < 3 hours/day displayed higher objectively monitored values for a number of physical activity indicators *and* less sedentary time than those reporting ≥ 3 hours/day. Finally, after categorizing participants based upon their cross-classified responses for self-reported MVPA, UODA, and LTSB, stark differences in the daily volume of ambulatory behavior were apparent among the eight combinations of activity classifications. Specifically, the most active adult combination (≥ 150 minutes/week of self-reported MVPA, “stand, walk, lift, or carry” UODA, and < 3 hours/day of LTSB) accumulated more than twice as many steps/day (4,403 steps/day more) as the least active combination (< 150 minutes/week of self-reported MVPA, “mostly sitting” UODA, and ≥ 3 hours/day of LTSB).

Until recently, self-report questionnaires served as the primary means to assess physical activity during the last 50 years [20]. Contemporary technological advancements have prompted a shift away from self-report methods in favor of objective monitoring (e.g., accelerometers, pedometers, multi-sensor devices) for physical activity assessment. Previous analyses of the 2003–2004 and 2005–2006 NHANES have demonstrated a substantial disconnect between self-reported and objectively monitored physical activity [7,21], as self-reported time spent in MVPA was substantially higher on average than objectively monitored estimates of MVPA accumulated in modified 10 minute bouts. Results presented herein agree with these findings as participants self-reporting compliance with MVPA guidelines (≥ 150 minutes/week) only accumulated a little more than 8 minutes/day of MVPA in modified 10 minute bouts or approximately 57 minutes/week. This continues to support the contention that self-reported physical activity provides poor estimates of the absolute amount of physical activity [22]. Despite this, current physical activity recommendations are still largely based on epidemiological evidence supporting the health benefits of physical activity, and specifically MVPA, as measured by self-report [23-26]. Results presented here show that, on average, adults who reported meeting current physical activity guidelines were more active across a range of physical activity indicators than those who did not meet the guidelines, in terms of objectively monitored behavior (but did not differ on sedentary behavior). Greater levels of most objectively monitored physical activity indicators among those reporting compliance with current physical activity guidelines were particularly apparent for all volume, rate, and time indicators of physical activity (except incidental movement, sporadic movement, and low intensity activity). Compliance to the waking-hour protocol did not appear to affect conclusions as wear time did not differ between the two self-reported MVPA categories. The lack of significant differences in non-movement, sedentary time, and breaks in sedentary time between self-reported MVPA groupings is consistent with previous notions that sedentary behavior and MVPA-related physical activity are distinct behaviors, with distinct determinants [27]. Thus, asking individuals about their MVPA does not appear to be useful in characterizing sedentary behavior habits. Incidental movement, sporadic movement, and low intensity activity were also not significantly different between categories of self-reported MVPA. This may be due to the proximity of incidental movement, sporadic movement, and low intensity activity (1–19 steps/minute, 20–39 steps/minute, and 100–499 activity counts/minute, respectively) on the objectively monitored scale to non-movement and sedentary time (0 steps/minute and < 100 activity counts/minute, respectively), in terms of movement intensity, providing further evidence that self-reported MVPA queries are of limited use in describing activity within these lower intensity ranges as well.

As we have pointed out previously [16], American adults spend the majority of their daily time engaged in sedentary behaviors or physical activities of less than moderate intensity. Furthermore, evidence suggests there is a strong and inverse relationship between these two types of behavior (sedentary behavior and light intensity physical activity) [28]. In contrast, Americans spend little time engaged in MVPA [7,16,21], which represents a very small proportion of an individual’s day. Moreover, evidence suggests that time spent in objectively monitored MVPA is not strongly associated with sedentary time or light intensity physical activity [29]. As such, it is not surprising that the relatively small, yet significant, difference in objectively monitored MVPA observed between categories of self-reported MVPA (< 150 vs. ≥ 150 minutes/week) in this investigation was not associated with any significant differences in sedentary time or physical activity at the lower end of the intensity spectrum (e.g., incidental movement, sporadic movement, and low intensity physical activity).

Daily occupation-related energy expenditure has decreased more than 100 calories over the last 50 years in the U.S. [30]. Societal changes in UODA resulting in pervasive replacement of movement with non-movement are believed to be driving this shift [30]. Our analysis of the NHANES data demonstrates that most accelerometer-derived variables were significantly different with regard to self-described UODA as “mostly sitting” vs. “stand, walk, lift, or carry,” with notable exceptions only amongst specific time indicators of activity. Similar to the MVPA analyses, wear time did not differ between the two self-described UODA categories; it therefore cannot be considered an important confounding variable explaining this relationship. The lack of UODA-related significant differences observed for the specific time indicators of brisk walking, faster locomotion, vigorous intensity activity, and moderate-to-vigorous intensity activity accumulated in modified 10 minute bouts is in keeping with the notion that estimates of sedentary time are independent of estimates of time spent in higher intensity and structured activities [28]. Somewhat more surprising is the fact that incidental movement (operationalized as 1–19 steps/minute) was not significantly different between polar UODA categories, whereas non-movement (0 steps/minute) was significantly different. Sedentary time (< 100 activity counts/minute) was also significantly different between UODA categories, indicating the primary source of this difference was due to discrepant non-movement rather than incidental movement (that may also be captured in the more relaxed definition of sedentary time using a less restrictive range of activity counts/minute). It is apparent that a simple question that quickly categorizes self-reported sitting vs. non-sitting behaviors during what is typically conceived as the most productive part of the day is sensitive to all volume and rate indicators of physical activity, sedentary time (especially that indicative of non-movement), and breaks in sedentary time.

Similar findings were apparent for self-reported LTSB dichotomized at the 3 hours/day threshold. Again, wear time did not appear to influence the results. In addition, and as expected [28], this question, which focuses on time spent in screen-based sedentary behaviors, did not differentiate the two categories with regard to time spent in brisk walking, faster locomotion, vigorous intensity activity, or moderate-to-vigorous intensity activity accumulated in modified 10 minute bouts. The lack of an association between self-reported television watching and time spent in leisure-time physical activity has been previously reported [31]. All other volume, rate, and time indicators were significantly different between LTSB categories. LTSB in this analysis included both television watching and leisure-time computer use, however, television watching is considered the more prevalent behavior [32].

This is the first investigation to evaluate levels of objectively monitored activity throughout cross-classifications of self-reported MVPA, UODA, and LTSB in a nationally representative sample of U.S. adults. Not surprisingly, those adults who reported being least physically active and most sedentary across all self-reported categories (< 150 minutes/week of MVPA, “mostly sitting” UODA, and ≥ 3 hours/day of LTSB) accumulated the fewest daily steps, while those who reported being most physically active and least sedentary (≥150 minutes/week of MVPA, “stand, walk, life, or carry” UODA, and < 3 hours/day of LTSB) had the highest daily step totals. The extremely low levels of ambulatory activity demonstrated by the least active and most sedentary category (≈ 3,500 steps/day) is indicative of minimal amounts of daily activity and representative of the “sedentary lifestyle index” (≤ 5,000 steps/day) [33-35]. Interestingly, adults in the most physically active and least sedentary grouping had daily step accumulations (≈ 7,950 steps/day) within the minimal range of daily steps (7,000-8,000 steps/day) indicative of free-living physical activity and compliance with current public health guidelines for MVPA [36].

Sisson and colleagues [9] previously examined UODA and LTSB in relation to risk of metabolic syndrome among U.S. adults in the 2003–2004 and 2005–2006 NHANES. Their findings indicated that UODA was not associated with metabolic syndrome in either men or women [9]. However, regardless of physical activity level, adult men accumulating ≥ 3 hours/day of LTSB had higher odds of metabolic syndrome compared to those accumulating < 3 hours/day, while physically inactive women (< 150 minutes/week of MVPA) who accumulated ≥ 3 hours/day of LTSB also had higher odds of metabolic syndrome in comparison to those accumulating < 3 hours/day. Our results demonstrate that further cross-classification of individuals according to their combined self-reported MVPA, UODA, and LTSB depicts a wider range of ambulatory activity (3,532-7,935 steps/day) than single categorizations for self-reported MVPA, UODA, or LTSB responses (widest range – UODA; 4,909-7,035 steps/day). As such, when studying disease risks in relation to self-reported physical activity and/or sedentary behavior, it may be beneficial to cross-classify individuals according to multiple and combined physical activity and sedentary behavior criteria to gain a better understanding of the relationships between these behaviors and specific disease outcomes.

A main strength of this study is its use of a large, nationally-representative sample of non-institutionalized adults in the U.S. Other strengths include the use of an objective measure of physical activity and sedentary behavior (accelerometer), as well as the use of a self-report physical activity questionnaire that assessed physical activity and sedentary behaviors specific to transportation, household/domestic tasks, and leisure-time activities. Because frequency and duration of activities were asked for each question, participants did not have to mentally sum varying frequency and duration values for different activities performed in different contexts, as is sometimes required by other self-report questionnaires asking global questions about how much moderate or vigorous activity was performed in the previous week.

This study has several limitations that must be acknowledged. As previously noted, self-report methods generally provide poor estimates of the absolute amount of physical activity [22]. This may be attributable to the cognitive complexity involved in recalling such behavior [37], as respondents are required to answer queries using terms which are often unfamiliar, ambiguous, or vague (e.g., “physical activity”, “moderate”, “vigorous”, etc.). Additionally, the NHANES question assessing transportation-related MVPA did not specifically ask about activities lasting at least 10 minutes in duration, even though values from this query were counted toward adults’ weekly physical activity duration in order to assess compliance with current physical activity guidelines. This is problematic because physical activity should be completed in bouts lasting at least 10 minutes in duration in order to be counted toward the 150 minutes/week of MVPA threshold as specified in current physical activity guidelines [8]. Due to the noted limitations of self-reported physical activity data, in general, and specifically in the NHANES protocol, the use of self-report when categorizing individuals as meeting or not meeting current physical activity guidelines in this study can be questioned. However, we chose this approach to remain consistent with previous investigations using similar methods to evaluate self-reported MVPA from the 2003–2004 and 2005–2006 NHANES [7,21].

Another limitation of this study worth noting is that the NHANES question we used to represent UODA is not strictly occupational in nature. Specifically, participants were asked to choose a statement that best described their usual daily activities while given examples that such activities could include work, housework (if a homemaker), attending classes (if a student), and other typical daily activities (if retired or unemployed). Given this, it is likely that some of the screen-based sedentary behaviors captured in the LTSB measure may also be represented in the UODA variable, and as such there may be some redundancy between the two variables. An additional limitation is that the NHANES queries we used to characterize LTSB only focused on screen-based sedentary behaviors, including television viewing and computer usage, which cannot adequately capture all leisure-time sedentary behaviors, such as those behaviors which are not screen-based, for example, reading a book or riding in a car. However, it is logical to measure screen-based sedentary behaviors as a starting point since television viewing by itself is the most commonly reported sedentary behavior in the U.S., comprising more than half of all self-reported leisure-time among participants ages ≥ 15 years [38].

## Conclusions

In summary, data derived from U.S. adults suggests that, on average, those who report higher levels of MVPA (≥ 150 minutes/week) engage in more physical activity (by a range of different indicators), but not less sedentary time, than those reporting lower levels of activity (< 150 minutes/week of MVPA). Moreover, adults who characterize the productive part of their day as engaging in substantial amounts of sedentary behavior (“mostly sitting” UODA or ≥ 3 hours/day of LTSB) are, on average, accumulating more objectively measured sedentary time and less physical activity (across a range of indicators) than those reporting being less sedentary (“stand, walk, life, or carry” UODA or < 3 hours/day of LTSB). Further stratifying adults based upon cross-classified responses for self-reported MVPA, UODA, and LTSB depicts a greater range of physical activity levels than simple classifications relying on a single self-reported capture of habitual behavior in any one category. Future research is needed to determine if these combined categorizations of individuals by queries assessing MVPA, UODA, and LTSB are also sensitive to theoretically associated health risk factors.

## Competing interests

The authors have received no funding for this work and declare no conflicts of interests.

## Authors’ contributions

JMS conducted the data treatment, performed the statistical analysis, and led the data presentation. WDJ assisted with the data treatment, statistical analysis, and data presentation. CT-L conceived the study. All authors contributed to the interpretation of results and participated in the drafting and critical revision of the manuscript. All authors read and approved the final manuscript.

## Supplementary Material

Additional file 1: Table S1 Means for accelerometer-determined variables among cross-classifications of self-reported MVPA, UODA, and LTSB in 2005–2006 NHANES adults. Click here for file
