# Supplementary material for: Adult self-reported and objectively monitored physical activity and sedentary behavior: NHANES 2005–2006
Source: Int J Behav Nutr Phys Act. 2013 Nov 11;10:126. doi: 10.1186/1479-5868-10-126 (PMC3828579; doi:10.1186/1479-5868-10-126)
Supplement: Additional file 1: Table S1 — Means for accelerometer-determined variables among cross-classifications of self-reported MVPA, UODA, and LTSB in 2005–2006 NHANES adults. [file 1479-5868-10-126-S1.pdf]

Additional file 1: Table S1. Means for accelerometer-determined variables among cross-classifications of self-reported MVPA, UODA, and LTSB in 2005-2006 NHANES adults.

	MVPA							
	< 150 minutes/week				≥ 150 minutes/week			
	UODA				UODA			
	Mostly sitting		Stand, walk, lift, or carry		Mostly sitting		Stand, walk, lift, or carry	
	LTSB		LTSB		LTSB		LTSB	
**Variable**	≥ 3 hours/day	< 3 hours/day	≥ 3 hours/day	< 3 hours/day	≥ 3 hours/day	< 3 hours/day	≥ 3 hours/day	< 3 hours/day
N	347	184	631	574	160	158	806	865
Accelerometer wear time (hours/day)	13.6 (0.1)	13.8 (0.2)	14.1 (0.1)	14.2 (0.1)	14.0 (0.1)	14.2 (0.1)	13.9 (0.1)	14.2 (0.1)
**Volume indicators**								
Steps (steps/day)								
Uncensored steps/day	6,034 (243)	7,764 (227)	9,175 (154)	10,346 (162)	8,562 (316)	9,180 (303)	9,932 (149)	11,235 (212)
Censored steps/day	3,532 (206)	4,805 (175)	5,979 (150)	6,849 (163)	5,580 (269)	6,472 (231)	6,837 (149)	7,935 (173)
Activity counts (counts/day)								
Activity counts/day	163,631 (7,520)	210,626 (6,772)	250,425 (4,923)	279,966 (6,369)	233,809 (10,017)	268,609 (9,578)	276,521 (5,641)	318,436 (4,776)
**Rate indicators**								
Steps (steps/minute)								
Uncensored steps/minute	7.3 (0.3)	9.3 (0.3)	10.9 (0.2)	12.1 (0.2)	10.2 (0.3)	10.7 (0.3)	11.9 (0.1)	13.2 (0.2)
Censored steps/minute	4.3 (0.3)	5.7 (0.2)	7.1 (0.2)	8.0 (0.2)	6.6 (0.3)	7.5 (0.3)	8.2 (0.1)	9.3 (0.2)
Peak 1-minute cadence	84.1 (1.8)	92.4 (1.3)	95.3 (1.0)	100.0 (0.9)	102.9 (2.1)	111.3 (2.0)	102.1 (0.9)	107.5 (0.8)
Peak 30-minute cadence	53.7 (1.3)	61.8 (1.1)	65.3 (1.0)	69.6 (0.7)	72.4 (2.5)	82.1 (2.4)	74.1 (1.1)	79.0 (1.0)
Activity counts (counts/minute)								
Activity counts/minute	198.1 (9.2)	251.5 (7.9)	297.3 (6.3)	328.8 (8.2)	277.8 (11.6)	312.8 (10.8)	330.0 (5.5)	372.8 (4.7)
**Time indicators**								
Cadence (minutes/day)								
Non-movement	356.3 (10.5)	305.4 (7.8)	293.9 (6.0)	263.4 (4.3)	312.6 (14.2)	324.5 (9.2)	286.7 (3.1)	265.2 (4.1)
Incidental movement	361.0 (4.7)	391.2 (5.7)	383.1 (4.0)	399.7 (4.0)	383.7 (11.2)	380.5 (8.3)	372.5 (2.7)	391.0 (2.9)
Sporadic movement	65.6 (2.9)	85.7 (3.0)	104.3 (1.8)	116.0 (2.3)	84.4 (3.3)	84.1 (3.8)	103.5 (2.1)	113.9 (2.5)
Purposeful steps	20.7 (1.6)	29.6 (1.4)	37.4 (1.1)	43.3 (1.0)	32.1 (1.8)	32.6 (1.7)	39.9 (0.8)	46.6 (1.2)
Slow walking	7.5 (0.6)	10.5 (0.6)	14.1 (0.6)	17.2 (0.6)	12.9 (1.2)	13.4 (0.6)	16.1 (0.4)	19.4 (0.8)
Medium walking	4.0 (0.3)	4.9 (0.3)	6.5 (0.4)	7.5 (0.3)	6.5 (0.6)	7.7 (0.5)	8.1 (0.5)	9.4 (0.4)
Brisk walking	2.4 (0.3)	3.1 (0.3)	3.3 (0.3)	3.7 (0.2)	5.3 (0.9)	7.6 (0.8)	6.0 (0.5)	6.4 (0.6)
Faster locomotion	0.4 (0.1)	0.6 (0.2)	0.5 (0.1)	0.6 (0.1)	2.3 (0.5)	4.2 (0.6)	1.6 (0.2)	2.5 (0.3)
Any movement	461.5 (7.9)	525.6 (7.5)	549.2 (4.2)	587.9 (5.3)	527.3 (14.1)	530.1 (12.5)	547.7 (4.7)	589.2 (5.2)
Non-incidental movement	100.5 (5.2)	134.4 (4.8)	166.1 (3.1)	188.3 (3.7)	143.5 (5.3)	149.5 (5.8)	175.1 (2.7)	198.2 (4.4)
Activity intensity (minutes/day)								
Sedentary time	551.2 (10.1)	503.9 (8.9)	479.9 (6.9)	455.9 (5.5)	513.9 (11.8)	527.1 (7.6)	469.0 (4.2)	455.3 (4.9)
Low intensity	168.6 (3.3)	197.2 (3.9)	204.7 (2.6)	217.5 (3.5)	192.6 (7.3)	179.6 (5.5)	196.7 (2.4)	207.0 (2.4)
Light intensity	87.5 (4.3)	115.3 (3.6)	140.0 (2.4)	156.6 (3.7)	113.3 (4.3)	120.8 (5.1)	144.4 (2.7)	162.4 (2.9)
Lifestyle intensity	50.6 (3.0)	68.5 (2.6)	84.9 (1.7)	97.4 (2.7)	69.2 (3.2)	76.5 (3.5)	89.6 (1.9)	103.8 (2.1)
Moderate intensity	10.3 (0.9)	14.3 (1.1)	18.3 (1.1)	21.0 (1.0)	18.8 (1.8)	24.6 (1.4)	23.3 (1.1)	28.4 (1.2)
Vigorous intensity	0.3 (0.1)	0.3 (0.1)	0.3 (0.1)	0.4 (0.1)	1.2 (0.5)	2.4 (0.7)	1.0 (0.2)	1.3 (0.2)
Moderate-to-vigorous intensity	10.5 (0.9)	14.6 (1.2)	18.6 (1.1)	21.4 (1.0)	20.0 (2.0)	27.0 (1.5)	24.3 (1.2)	29.7 (1.2)
Moderate-to-vigorous intensity in 10 minute bouts	1.5 (0.2)	3.3 (0.6)	3.5 (0.4)	3.8 (0.4)	6.5 (1.4)	10.6 (1.0)	7.1 (0.6)	9.0 (0.8)
**Breaks in sedentary time**								
Transitions/day	83.7 (1.0)	92.7 (1.2)	91.3 (1.0)	95.3 (1.1)	91.8 (2.0)	91.2 (1.9)	89.3 (0.6)	94.0 (0.7)

Values weighted to provide nationally representative estimates and presented as mean (SE). MVPA = moderate-to-vigorous physical activity; UODA = usual occupational/domestic activity; LTSB = leisure-time sedentary behavior.
